# Supplementary material for: Fluorescent artificial receptor-based membrane assay (FARMA) for spatiotemporally resolved monitoring of biomembrane permeability
Source: Commun Biol. 2020 Jul 15;3:383. doi: 10.1038/s42003-020-1108-9 (PMC7363885; doi:10.1038/s42003-020-1108-9)
Supplement: Supplementary file 1 — Supplementary Information [file 42003_2020_1108_MOESM1_ESM.pdf]

Supplementary Figures

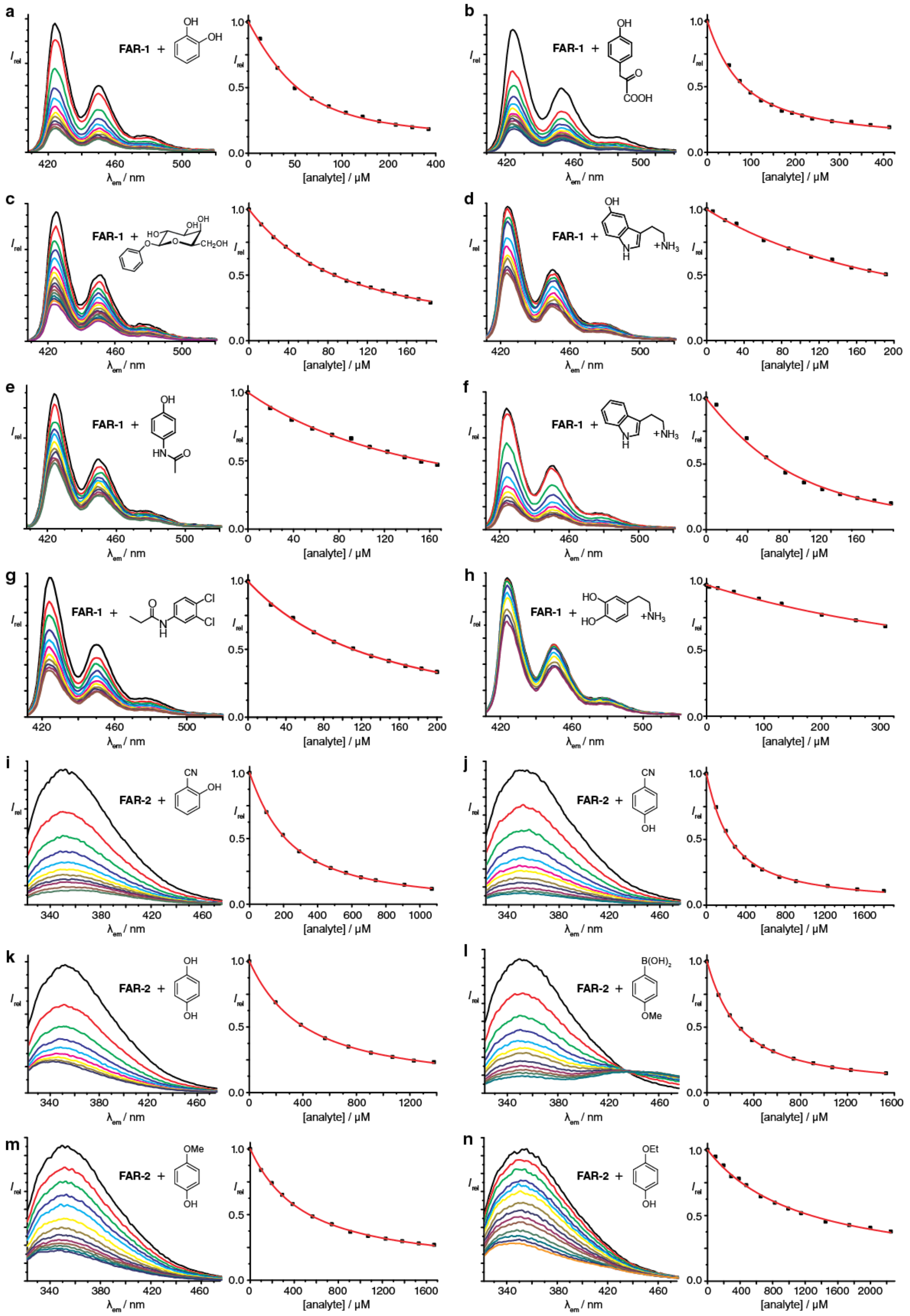

Supplementary Figure 1 | Emission spectra and fittings (1:1 binding model) for the titration of **FAR-1** (λ_{ex} = 400 nm, λ_{em} = 450 nm) with (a) catechol, (b) 4-hydroxyphenylpyruvic acid, (c) phenyl-β-D-galactopyranoside, (d) serotonin, (e) paracetamol, (f) tryptamine, (g) propanil, (h) dopamine. Emission spectra and fittings (1:1 binding model) for the titration of **FAR-2** (λ_{ex} = 310 nm, λ_{em} = 370 nm) with (i) 2-hydroxybenzonitrile, (j) 4-hydroxybenzonitrile, (k) hydroquinone, (l) *p*-anisylboronic acid, (m) 4-methoxyphenol and (n) 4-ethoxyphenol. All titrations were carried out in homogenous solution, 10 mM HEPES buffer, pH 7.

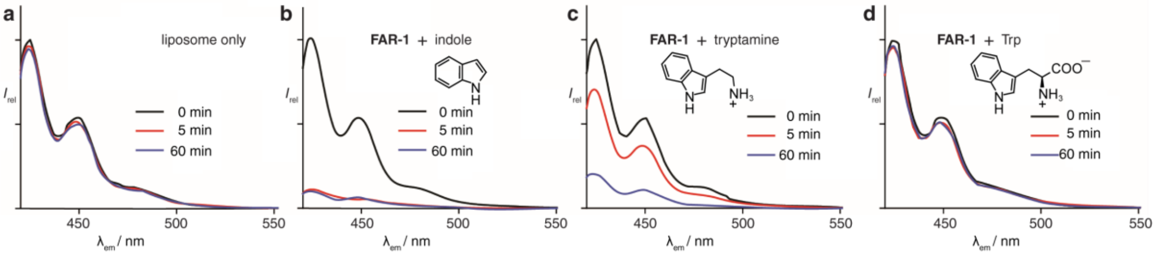

Supplementary Figure 2 | FARMA assay recorded on a microplate reader (96-well plates) with **FAR-1**. (a) In the absence of analyte, no significant change in the emission intensity of **FAR-1** occurred during 60 min, demonstrating their stability in the microplate wells. (b-d) Upon addition of different analytes, fluorescence readings at 0, 5 and 60 minutes (normalized to initial intensities) revealed a rapid decrease in fluorescence for (b) indole and (c) tryptamine, but no significant change for (d) tryptophan (Trp). Note that the 5-min (red) and 60-min (blue) traces for indole and tryptophan are essentially superimposed. The results are consistent with the permeability of the same analytes obtained from quartz cuvette-based assays, *i.e.*, instantaneous permeation of indole, slow permeation of tryptamine, and no permeation of Trp.

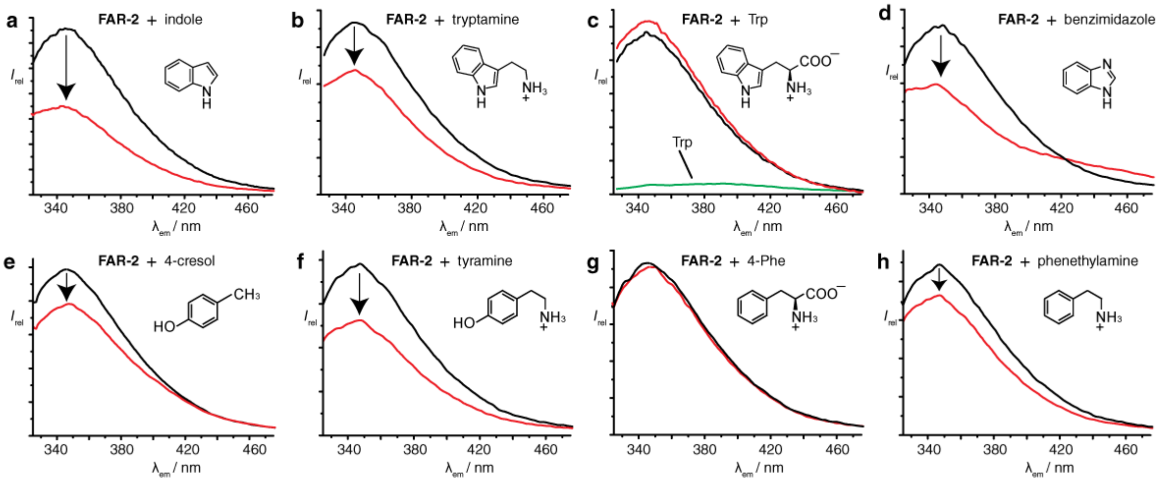

Supplementary Figure 3 | FARMA assay recorded in quartz cuvettes with **FAR-2** and selected analytes, (a) indole (10 µM), (b) tryptamine (8 µM), (c) Trp (20 µM), (d) benzimidazole (8 µM), (e) 4-cresol (20 µM), (f) tyramine (10 µM), (g) Phe (20 µM), and (h) phenethylamine (10 µM). The spectra (λ_{ex} = 310 nm) were recorded prior to analyte addition (black trace) and after analyte addition (red trace), once the system had equilibrated. Note that the apparent slight increase of the emission for the FARMA experiment with Trp (panel c) is due to the weak autofluorescence of Trp at 310 nm excitation (green trace).

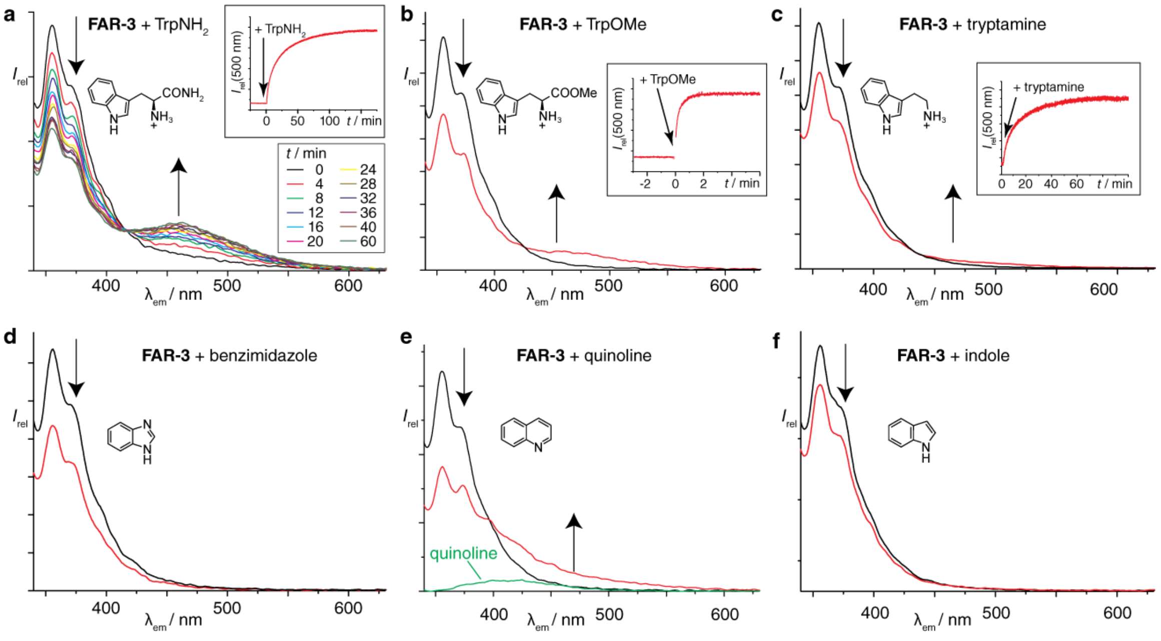

Supplementary Figure 4 | FARMA assay with **FAR-3** and representative analytes, each at 16 µM, (a) $TrpNH_2$, (b) TrpOMe, (c) tryptamine, (d) benzimidazole, (e) quinoline, and (f) indole. The spectra (λ_{ex} = 330 nm) were recorded prior to analyte addition (black trace) and after analyte addition (red trace), once the system had equilibrated. In some cases, both the quenching of the emission band of **FAR-3** around 370 nm, but also the appearance of an excimer band from 450-550 nm is observed. The insets show the time-resolved monitoring of the relative emission intensity at 500 nm. Panel c shows also the weak autofluorescence of quinoline at 330 nm excitation under the same measurement conditions (green trace).

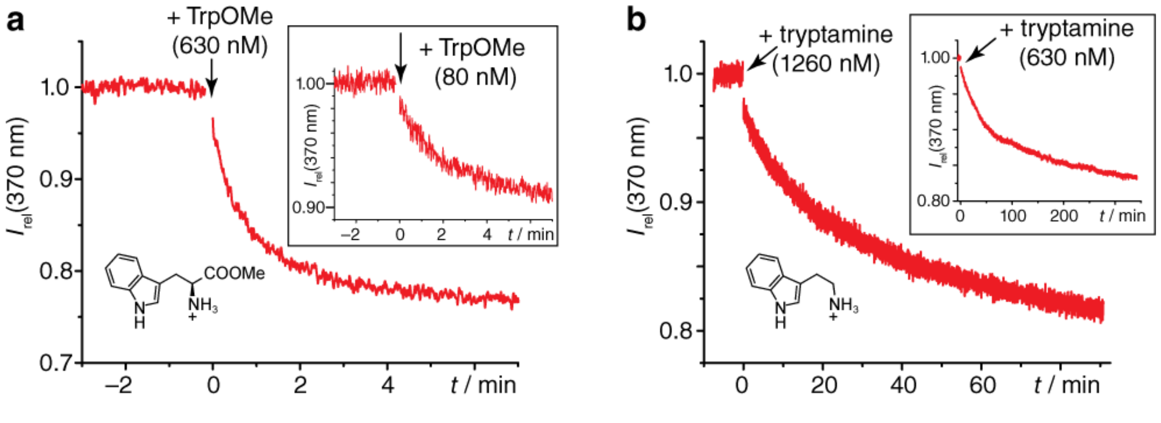

Supplementary Figure 5 | FARMA assay with **FAR-2** (λ_{ex} = 310 nm) and (a) TrpOMe and (b) tryptamine at different concentrations in order to demonstrate the high sensitivity of the method.

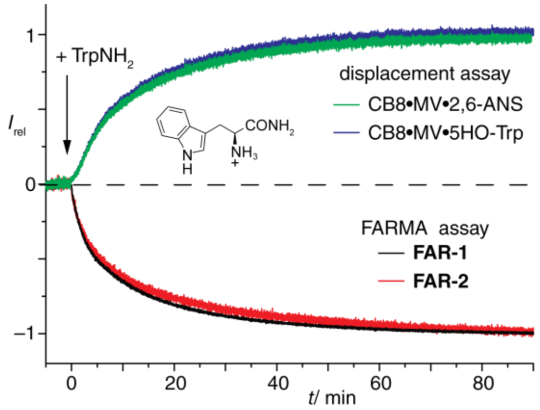

Supplementary Figure 6 | Translocation monitoring of $TrpNH_2$ (aq. stock, 16 µM) with **FAR-1**- and **FAR-2**-loaded liposomes, and with two membrane-encapsulated indicator displacement ensembles (blue and green).

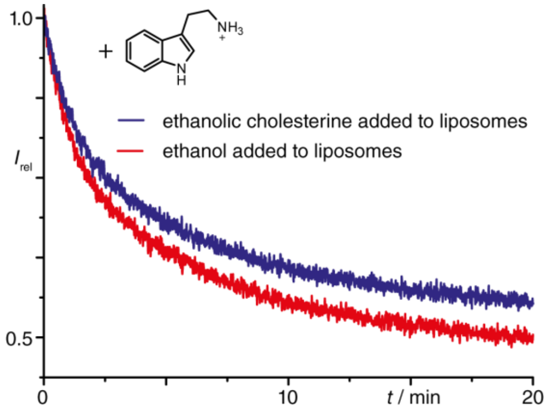

Supplementary Figure 7 | Translocation monitoring of tryptamine (aq. stock, 16 µM) with **FAR-2** loaded liposomes (1000 µL of the same aqueous liposome stock), which have been pre-treated for 20 min with 10 µL of a 10 mM ethanolic cholesterol solution, or with 10 µL of ethanol as the control.

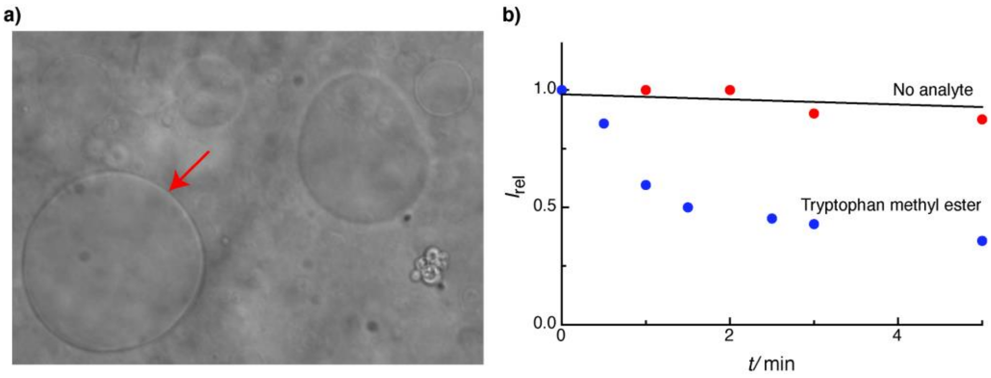

Supplementary Figure 8 | (**a**) Phase-contrast image of **FAR-1**-loaded GUVs. The red arrow points to the GUV, whose fluorescence we selected to follow after the addition of the analyte, tryptophan methyl ester (TrpOMe), see Fig. 3g in main text. (**b**) Normalized fluorescence intensities of GUVs in the absence and presence of TrpOMe monitored at different times (after addition). In the absence of TrpOMe no noticeable change in fluorescence intensity was observed, which further signifies that the decrease in intensity upon addition of TrpOMe cannot be attributed to photobleaching.

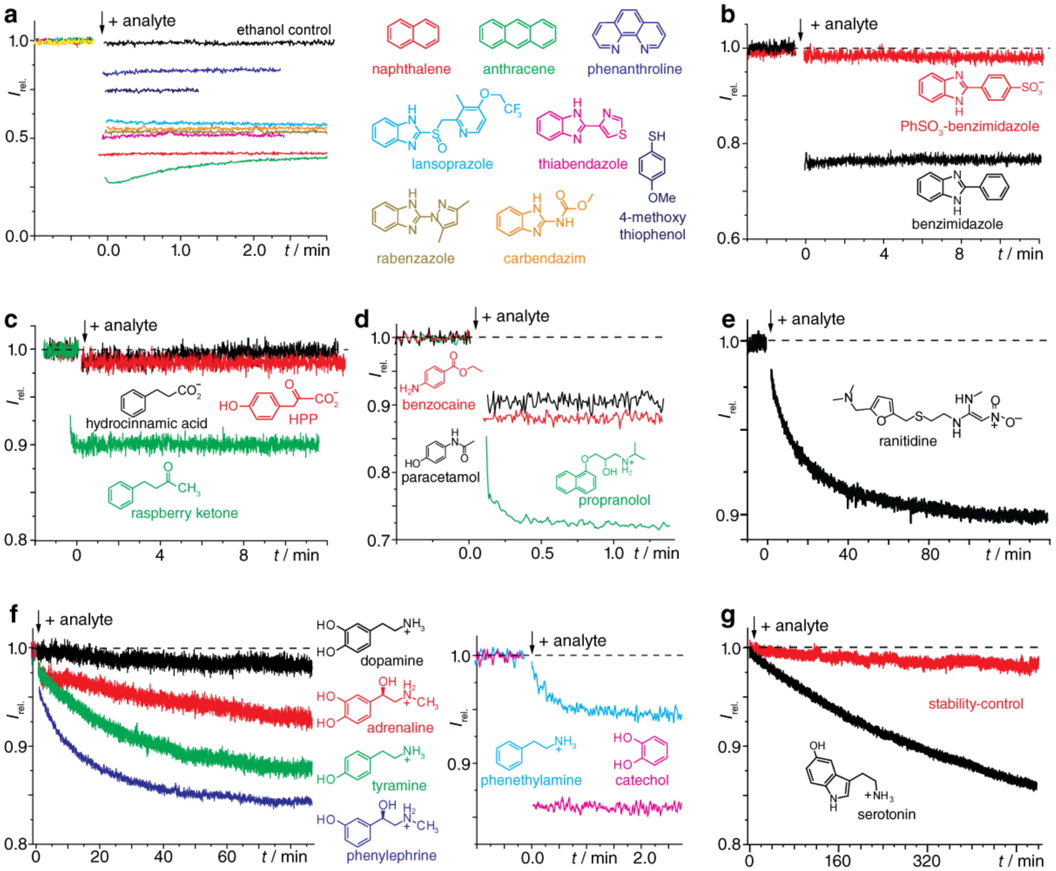

Supplementary Figure 9 | FARMA assay with **FAR-1** (λ_{ex} = 400 nm, λ_{em} = 450 nm) and representative analytes, each at 8 µM in 10 mM aqueous HEPES buffer, pH 7. (a) Hydrophobic aromatics that were titrated from an ethanolic stock solution and the corresponding blank control, (b-c) structurally related substances for investigating biomembrane permeability of non-charged *vs*. negatively charged species, (d-e) representative drugs, (f) catecholamine neurotransmitter and related substances, (g) slow permeation trace of the neurotransmitter serotonin and photostability control in the absence of analyte. The titration of the analytes was conducted from an aqueous stock with the exception of analytes shown in (a).

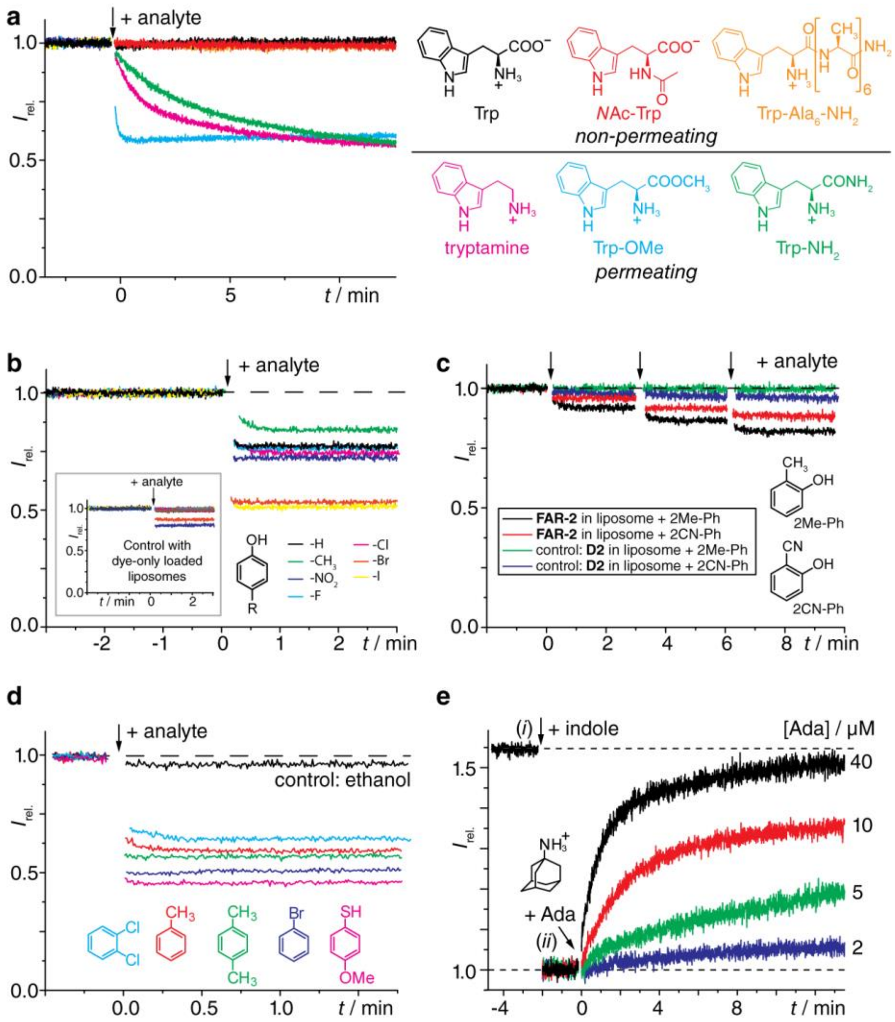

Supplementary Figure 10 | FARMA assay with **FAR-2** (λ_{ex} = 310 nm, λ_{em} = 370 nm) and representative analytes. (a) Trp and related species (each at 8 μM), traces were corrected for autofluorescence of Trp-species. (b) *p*-substituted phenols (8 μM), the inset shows the control experiments with D2 dye-only loaded liposomes. (c) Consecutive addition of aliquots (each step 8 μM) for weakly binding analytes. The control shows the addition of analyte to liposomes that were loaded only with dye D2. (d) Hydrophobic aromatics (80 μM) that were titrated from an ethanolic stock solution and the corresponding blank control (e) Dye-displacement variant with **FAR-2** to monitor the membrane-permeation of aliphatic adamantylamine (Ada) via (*i*) addition of 10 μM of rapidly permeating indole, causing emission quenching of **FAR-2**. (*ii*) Addition of Ada, causing a displacement of both indole and the disassembly of **FAR-2**, such that the emission of free dye D2 is observed. See further below for a detailed description of the dye displacement approach. The experiments were carried out in 10 mM aqueous HEPES buffer, pH 7.

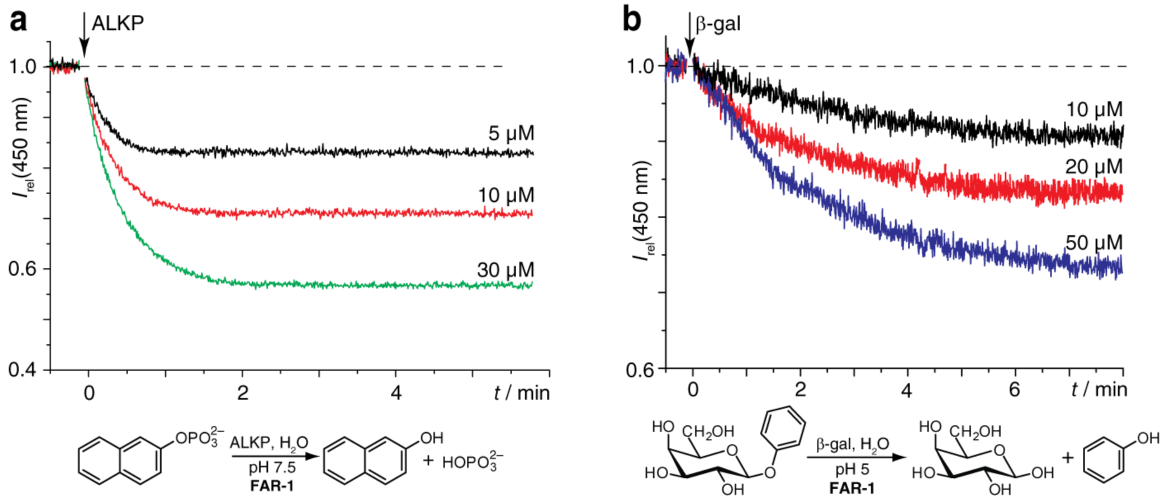

Supplementary Figure 11 | Monitoring of enzymatic reactions with **FAR-1** in homogenous solution, i.e., in the absence of a protective membrane. See Fig. 4 in the main text for the corresponding FARMA assays.

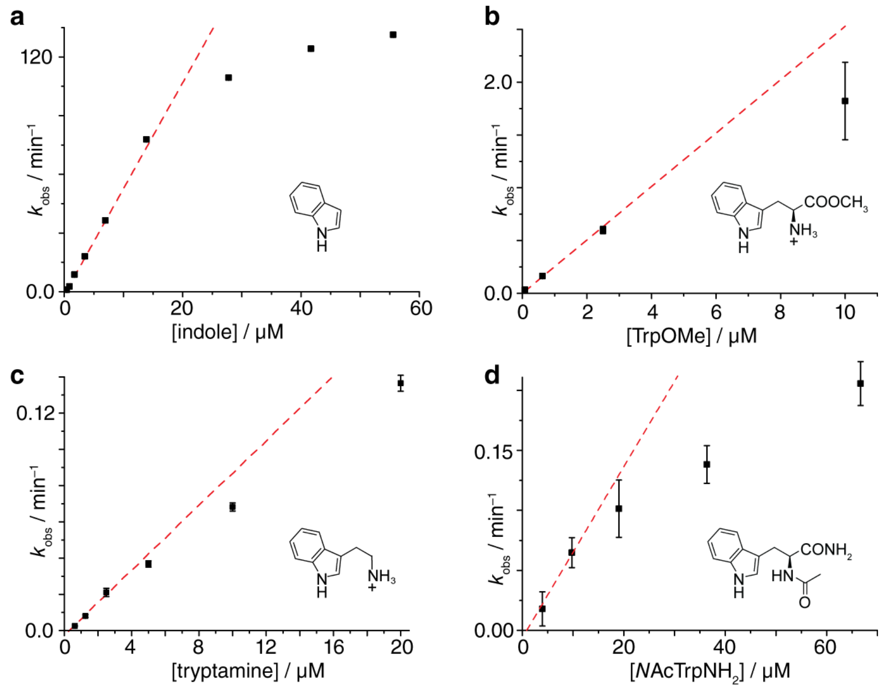

Supplementary Figure 12 | Observed concentration dependent permeation rate of (a) indole, (b) TrpOMe, (c) tryptamine and (d) *N*AcTrpNH2 into **FAR-2**-loaded liposomes (λ_{ex} = 310 nm, λ_{em} = 370 nm). For indole, the rates were extracted from monoexponential fits of the time-resolved fluorescence traces. For the other analytes, the initial rate method was used because of the observed non-exponential shape of the kinetic traces. The error bars denote the estimated fitting errors of the initial rates. The dotted line is a fit of the data points were the rate-concentration dependence is approximately linear. Note the saturation behavior at higher analyte concentrations.

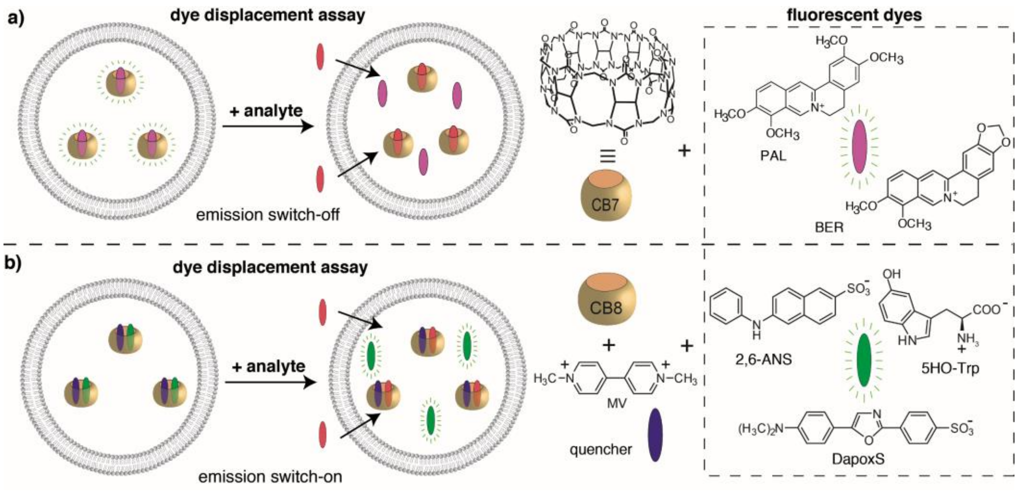

Supplementary Figure 13 | Graphical representation of membrane assays using dye displacement for signaling. Diffusion of analytes through a lipid membrane is detected by displacement of the dye from (a) a host•dye or (b) a host•dye•quencher complex, leading to reduction or enhancement in emission intensity.

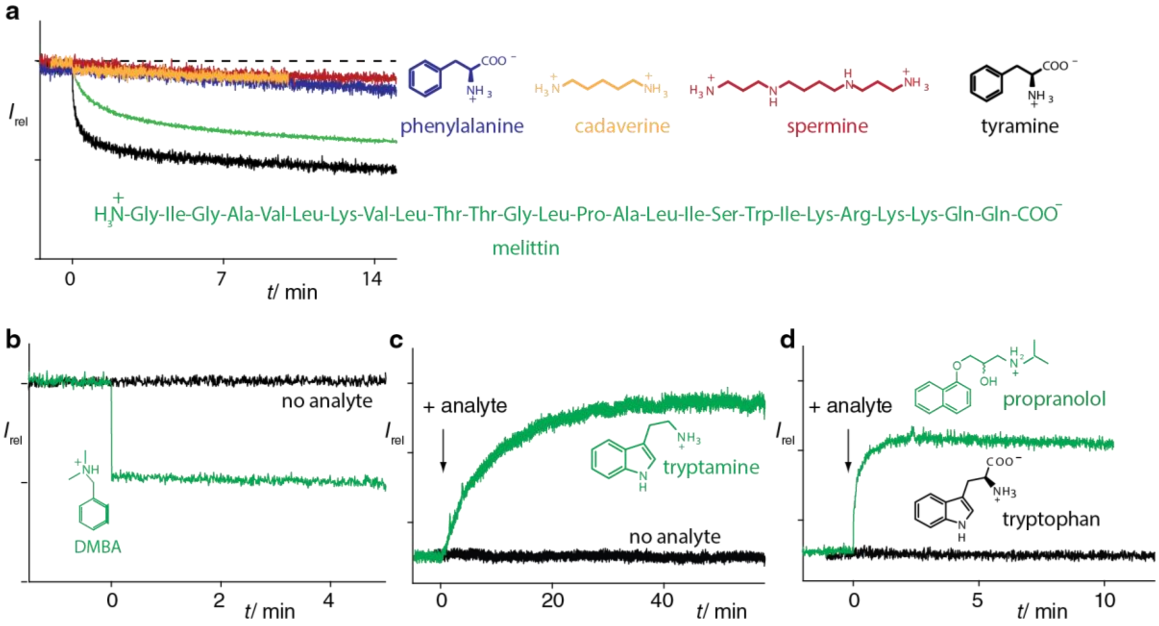

Supplementary Figure 14 | Membrane permeation monitoring of analytes by dye displacement. (a) Time-resolved changes in fluorescence intensity of CB7•BE-loaded liposomes (λ_{exc} = 347 nm, λ_{obs} = 500 nm) upon addition of the membrane-permeable analyte tyramine and membrane destabilizing peptide melittin. No significant fluorescence changes other than photobleaching of BE, verified by control experiments, is observed upon addition of spermine, cadaverine and phenylalanine, implying that these multiply charged species are membrane impermeable. (b) Evolution of fluorescence intensity after addition of dimethylbenzylamine (DMBA) to CB7•PAL-loaded liposomes (λ_{exc} = 347 nm, λ_{obs} = 500 nm). (c) Fluorescence response after addition of the slowly membrane permeable analyte tryptamine to liposomes loaded with CB8•MV•5OH-Trp (λ_{exc} = 310 nm, λ_{obs} = 337 nm). The black trace shows the control for the addition of the same volume of blank buffer. (d) Fluorescence response after addition of the membrane permeable analyte propranolol and of impermeable tryptophan to liposomes loaded with CB8•MV•2,6-ANS (λ_{exc} = 380 nm, λ_{obs} = 463 nm).

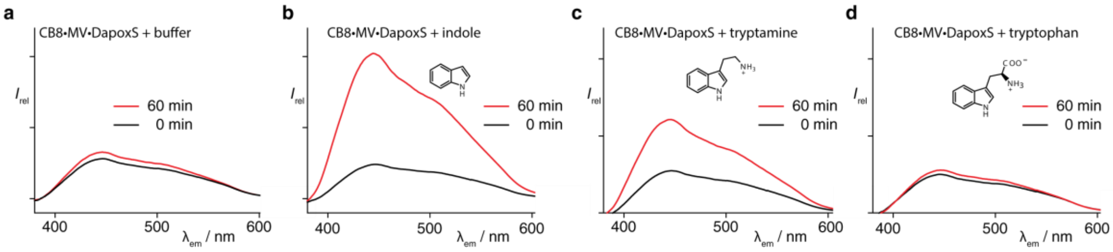

Supplementary Figure 15 | Dye displacement-based membrane permeation assay recorded on a microplate reader (96-well plates) with CB8•MV•DapoxS. (a) In the absence of analyte, no significant change in the emission intensity occurred during 60 min, demonstrating the stability of the chemosensing ensemble in the microplate wells. (b-d) Normalized fluorescence readings prior to analyte (16 µM) addition (0 min) and after analyte addition (60 min equilibration) revealed an increase in fluorescence for (b) indole and (c) tryptamine, but no significant change for (d) tryptophan (Trp).

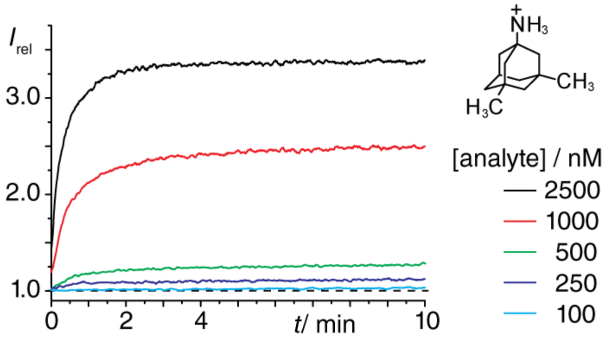

Supplementary Figure 16 | Dye-displacement-based membrane permeation assay for memantine (K_a for CB8 approx. 10^{11} M^{-1})[11, 12] with CB8•MV•5HO-Trp-loaded liposomes. Unlike the examples shown in Supplementary Figures 14c-d & 15 where the aromatic analyte displaced only the dye from the CB8•quencher•dye complex, in this example both the quencher and the dye are displaced from CB8 by the large, aliphatic guest memantine.

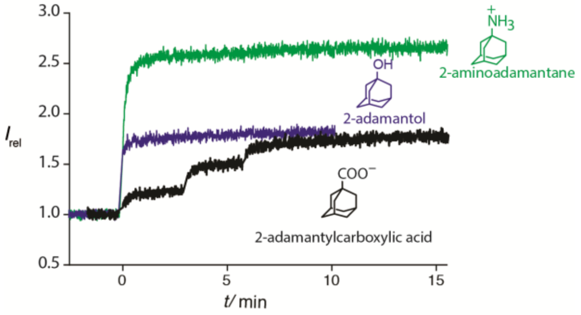

Supplementary Figure 17 | Addition of 50 µM adamantane derivatives to CB8•MV•5OHTrp-loaded liposomes. On account of the comparably small changes in relative fluorescence intensity for the negatively charged analyte 2-adamantylcarboxylate, multiple additions were performed to undoubtedly verify its membrane-permeation. See further below for a detailed description of the dye displacement approach.

Supplementary Tables

Supplementary Table 1 | Quenching efficiencies and binding constants (error estimate ≤ 20%) for the formation of FAR•analyte complexes in 10 mM HEPES buffer (pH 7), determined by fluorescence titrations in homogenous solution.

FAR	analyte	QE[a]	K_a / (10^3 M^{-1})[b]
**FAR-1**	1,2-dihydroxybenzene	>0.9	20
**FAR-1**	1,4-benzoquinone	>0.9	0.5
**FAR-1**	1,4-dihydroxybenzene	>0.9	13
**FAR-1**	4-ethoxyphenol	>0.9	1
**FAR-1**	4-ethylphenol	0.6	1.2
**FAR-1**	4-hydroxyphenylpyruvic acid (HPP)	>0.9	17
**FAR-1**	5HO-Trp	>0.9	7.7
**FAR-1**	adrenaline	- - -	<1
**FAR-1**	aniline	0.9	33
**FAR-1**	benzene	<0.3	4
**FAR-1**	benzimidazole	0.8	7.5
**FAR-1**	ceftazidime	- - -	<1
**FAR-1**	chloramphenicol	- - -	<1
**FAR-1**	dopamine	>0.9	0.10
**FAR-1**	homogentisic acid (HGA)	>0.9	0.14
**FAR-1**	hydroquinone	>0.9	13
**FAR-1**	*N*Ac-Trp	>0.9	50
**FAR-1**	*N*Ac-TrpNH_2	>0.9	110
**FAR-1**	naphthalene	>0.9	>50
**FAR-1**	naproxen	0.5	2
**FAR-1**	*N*-phenylsuccinimide	>0.9	30
**FAR-1**	paracetamol	>0.9	7
**FAR-1**	Phe	0.5	5
**FAR-1**	phenol	>0.9	56
**FAR-1**	phenylethylamine	0.4	8
**FAR-1**	phenyl-β-D-galactopyranoside	>0.9	30
**FAR-1**	prochloraz	0.7	2.8
**FAR-1**	propanil	>0.9	8
**FAR-1**	propranolol	>0.9	3
**FAR-1**	quinoline	0.6	2.2
**FAR-1**	ranitidine	0.2	2
**FAR-1**	rotenone	0.7	4.8

sensing ensemble	analyte	QE[a]	K_a / (10^3 M^{-1})[b]
**FAR-1**	serotonin	>0.9	5
**FAR-1**	Trp	>0.9	150
**FAR-1**	$TrpNH_2$	>0.9	460
**FAR-1**	TrpOMe	>0.9	14
**FAR-1**	tryptamine	>0.9	50
**FAR-2**	2,6-dihydroxynapthalene	>0.9	72[c]
**FAR-2**	2,7-dihydroxynapthalene	>0.9	66[c]
**FAR-2**	2-cyanophenol	>0.9	5.8
**FAR-2**	2-naphthol	>0.9	140[c]
**FAR-2**	4-cyanophenol	>0.9	5.2
**FAR-2**	4-ethoxyphenol	>0.9	1.0
**FAR-2**	4-iodophenol	>0.9	4.1[c]
**FAR-2**	4-methoxyphenol	>0.9	2.4
**FAR-2**	*p*-anisylboronic acid	>0.9	4.8
**FAR-2**	aniline	>0.9	25
**FAR-2**	GlyGlyTrp	>0.9	4.1[c]
**FAR-2**	GlyTrpGly	>0.9	17[c]
**FAR-2**	hydroquinone	>0.9	2.9
**FAR-2**	indole	>0.9	180[c]
**FAR-2**	phenol	>0.9	15[c]
**FAR-2**	sesamol	>0.9	4.2[c]
**FAR-2**	sodium benzoate	n.a.	<<1
**FAR-2**	Trp	>0.9	34[c]
**FAR-2**	TrpGlyGly	>0.9	120[c]
CB8•MV	2,6-dihydroxynapthalene	n.a.	590[e]
CB8•MV	2,7-dihydroxynapthalene	n.a.	160[e]
CB8•MV	2-naphthol	n.a.	610[e]
CB8•MV	4-iodophenol	n.a.	13[e]
CB8•MV	GlyGlyTrp	n.a.	3.1[d]
CB8•MV	GlyTrpGly	n.a.	21[d]
CB8•MV	*N*Ac-$TrpNH_2$	n.a.	3.1[d]
CB8•MV	Phe	n.a.	5.3[d]
CB8•MV	phenol	n.a.	22[e]
CB8•MV	Trp	n.a.	43[d]
CB8•MV	TrpGlyGly	n.a.	130[d]
CB8•MV	TrpOMe	n.a.	63[d]

sensing ensemble	analyte	QE[a]	K_a / (10^3 M^{-1})[b]
CB8•MV	tryptamine	n.a.	54[d]
CB8•MV	Tyr	n.a.	2.2[d]
CB7	cadaverine	AO, >0.9[f]	4500[i]
CB7	*N,N*-dimethylbenzylamine(DMBA)	PAL, >0.9[f]	3040[g]
CB7	Phe	AO, >0.9[f]	170[j]
CB7	phenylethylamine	AO, >0.9[f]	6800[j]
CB7	tryptamine	DAPI, 0.8[f]	130[h]
CB7	tyramine	DAPI, 0.8[f]	3800[h]
CB7	vitamin B1	PAL, >0.9[f]	6920[g]
CB7	nicotine	BE, 0.9[f]	160[g]
CB7	spermine	BE, 0.9[f]	2170[g]
CB7	1-adamantanol	[k]	2.3×10^7
CB7	1-adamantylamine	[k]	1.7×10^{11}
CB7	1-adamantanecarboxylic acid	[l]	3.2×10^5
CB7	Tyr	[m]	22

[a] Quenching efficiency. [b] Obtained by nonlinear least-square fitting to a 1:1 binding model in 10 mM phosphate buffer, taken from ref. [13]. [c] From ref. [13], in 10 mM sodium phosphate buffer (pH 7). [d] From ref. [14], in 10 mM sodium phosphate buffer (pH 7), by isothermal titration calorimetry (ITC). Note that MV is not emissive. [e] From ref.[15], in 10 mM sodium phosphate buffer (pH 7), by ITC; for other representative binding constants of CB8•MV with aromatic compounds see the same reference. [f] From dye-displacement titrations; here, the quenching efficiency is reported as the total change in fluorescence intensity upon complete displacement of the dye from the host cavity, which is irrespective of the photophysical properties, *e.g.*, quenching ability, of the dye. [g] In 10 mM sodium phosphate buffer (pH 7), [h] From ref.[16]. [i] From ref.[17]. [j] From ref. [18]. [k] From ref.[19], by ITC. [l] From ref.[11], in 50 mM NaO_2CCD_3-buffered D_2O (pD 4.74). [m] From ref.[16], by ITC.

Supplementary Table 2 | Experimentally observed rates (k_{obs}) and apparent permeability coefficients (P_{app}) for permeation of non-charged, rapidly permeating aromatic analytes through liposomal POPC:POPS bilayer membranes (*r* ca. 100 nm), their corresponding octanol-water partition coefficients (log*P*), and van der Waals volumes (V_W); ordered from slowest (top) to fastest permeation rate.

entry	analyte	k_{obs}/s^{-1} [a]	log*P* [b]	V_w/Å^3 [c]
**1**	toluene	75	2.5	103
**2**	propanil	42	2.7	179
**3**	4CN-phenol	40	1.7	111
**4**	4NO_2-phenol	38	1.4	112
**5**	*p*-xylene	17	3.0	120
**6**	2CN-phenol	10	1.7	111
**7**	aniline	7.5	1.2	98
**8**	4CN-aniline	6.5	1.3	116
**9**	4F-aniline	4.4	1.4	100
**10**	4Cl-aniline	4.0	1.8	112
**11**	4Br-aniline	4.0	2.1	119
**12**	4I-aniline	3.1	2.6	127
**13**	4F-phenol	2.8	1.8	96
**14**	benzimidazole	2.7	1.2	112
**15**	phenol	2.7	1.6	93
**16**	indazole	2.4	1.4	112
**17**	4Cl-phenol	2.2	2.2	107
**18**	indole	1.8	1.6	117
**19**	4Br-phenol	1.6	2.5	115
**20**	*N*-phenylsuccinimide	1.1	0.87	175
**21**	4I-phenol	0.69	3.0	122
**22**	DMABN	0.68	2.4	150
**23**	pentafluoroaniline	0.51	2.0	111
**24**	4Me-phenol	0.40	2.1	110
**25**	2Me-phenol	0.33	2.1	110
**26**	4Et-phenol	0.24	2.6	127
**27**	phenethylamine	0.22	1.4	134
**28**	4*t*Bu-phenol	0.062	3.4	160

[a] Observed pseudo-unimolecular rates (k_{obs}), obtained by fitting of the time-resolved fluorescence intensities with a monoexponential decay function. Experiments were carried out at an analyte concentration of 40 µM (in the bulk) and a **FAR-2** concentration of ca. 500 µM in the liposome interior; 20% error (reproducibility). [b] log*P* (octanol) was obtained by using the software ChemDraw. [c] van der Waals volume of analytes was calculated by using the software HyperChem.

Supplementary Table 3 | Qualitative assessment of membrane permeability for more than 90 different organic compounds[a] investigated by the FARMA-assay with receptors FAR-1, FAR-2, and FAR-3, and, for comparison, investigations by the dye displacement signal generation strategy with the sensing ensembles (CB8•MV•2,6-ANS, CB8•MV•5HO-Trp, CB7•BE, and CB7•PAL).

analyte[a]	**FAR-1**[b]	**FAR-2**[c]	**FAR-3**[d]	CB8•MV• 2,6-ANS[e]	CB8•MV• 5HO-Trp[f]	CB7• BE[g]	CB7• PAL[h]
1,2-dihydroxybenzene (catechol)	+	+ [i]					
(*R*)-phenylephrine	+	+					
1-adamantylamine (1-ADA)				+	+		
1-adamantanecarboxylic acid (1-ADC)				+	+		
1-adamantanol				+	+		
1,2-dichlorobenzene		+ [k]					
1,4-benzoquinone	+ [k]						
1,4-dihydroxybenzene (hydroquinone)	+						
1,4-dimethylbenzene (*p*-xylene)		+ [k]					
1-naphthyl phosphate	–						
2-cyanophenol	+	+					
2-methylphenol (*o*-cresol)	+	+					
2-naphthol (2-NpOH)	+			+ [o]			
2-naphthyl phosphate	–						
2-naphthylsulfonamide	+			+ [o]			
2-phenylbenzimidazole (Ph-benzimidazole)	+			+			
2-phenylbenzimidazole-5-sulfonic acid (O_3SPh-benzimidazole)	–			–			
2,7-dihydroxynaphthalene (2,7-Np$(OH)_2$)	+			+ [o]			
3-phenylpropanoic acid (hydrocinnamic acid)	–	–					
3,5-dimethyladamantan-1-amine (memantine)		+		+	+		
4-(*N,N*-dimethyl amino) benzonitrile (DMABN)		+					
4-bromoaniline	+	+					
4-bromobenzene		+ [k]					
4-bromophenol	+	+ [n]					
4-chloroaniline	+	+					
4-chlorophenol	+	+ [n]					
4-cyanoaniline	+	+					
4-cyanophenol	+	+ [n]					

analyte[a]	**FAR-1**[b]	**FAR-2**[c]	**FAR-3**[d]	CB8•MV•<br>2,6-ANS[e]	CB8•MV•<br>5HO-Trp[f]	CB7•<br>BE[g]	CB7•<br>PAL[h]
4-ethoxyphenol	+						
4-ethylphenol	+	+ [n]					
4-fluoroaniline	+	+					
4-fluorophenol	+	+ [n]					
4-hydroxybenzyl acetone (raspberry ketone)	+						
4-hydroxyphenylpyruvic acid (HPPA)	−						
4-iodoaniline	+	+					
4-iodophenol	+	+ [n]					
4-methoxyphenol	+						
4-methoxythiophenol	+ [k]	+ [k]					
4-methoxyphenylboronic acid (*p*-anisylboronic acid)		+					
4-methylaniline	+						
4-methylphenol (*p*-cresol)	+	+ [n]					
4-nitrophenol	+	+ [n]					
4-*tert*-butylphenol		+ [n]					
5-hydroxytryptophan (5HO-Trp)	−				−		
adamantane				+			
adrenaline	−	−					
ampicillin		−					
aniline	+	+ [n]					
anthracene	+[k]						
bentazon		+[k]					
benzene		+ [k]					
benzimidazole	+	+	+				
bisphenol A	+ [i,k]	+ [k]					
cadaverine						−	−
carbendazim	+ [k]			+ [k]			
N,*N*-dimethylbenzylamine (DMBA)						+	+
dopamine	−	−					
ethyl 4-aminobenzoate (benzocaine)	+						
imidacloprid		+[k]					
indazole	+	+					
indole	+	+	+	+	+		
lansoprazole	+[k]						
melatonin	+ [n]			+ [n]			
melittin	+ [p]	+ [p]	+ [p]	+ [p]		+ [p]	+ [p]

analyte[a]	**FAR-1**[b]	**FAR-2**[c]	**FAR-3**[d]	CB8•MV• 2,6-ANS[e]	CB8•MV• 5HO-Trp[f]	CB7• BE[g]	CB7• PAL[h]
benzene		+ [k]					
benzimidazole	+	+	+				
methyl benzoate		+ [k]					
N-acetyl tryptophan (*N*Ac-Trp)	–	–					
N-acetyl tryptophan amide (*N*Ac-TrpNH$_2$)	+	+	+ [m]				
N-phenylsuccinimide	+						
naphthalene	+ [k]			+ [o]			
nicotine		+		+		+	
omeprazole	+ [k]						
paracetamol	+	+ [i]					
penicillin G		–					
pentafluoroaniline	+	+					
phenanthroline	+ [k]						
phenol	+	+					
phenyl-β-D-galactopyranoside	+	+					
phenylalanine (Phe)		–				–	–
phenylethylamine	+	+				+	+
primaquine	+			+	+		
propanil	+	+					
propranolol	+			+			
quinoline	+	+	+ [m]	+ [o]			
rabenzazole	+ [k]						
ranitidine	+						
serotonin	+	+		+			
somatostatin	–	–					
spermine						–	–
thiabendazole	+ [k]			+ [k]			
toluene		+ [k]					
tropicamide		+ [k]					
Trp-(Ala)$_6$-NH$_2$	–	–					
Trp-(Leu)$_6$-NH$_2$	–	–					
Trp-(Lys)$_6$-NH$_2$	–	–					
tryptamine	+	+	+ [m]	+	+	+	
tryptophan (Trp)	–	–		–	–		
tryptophan amide (TrpNH$_2$)	+	+	+ [m]	+	+		
tryptophan methyl ester (TrpOMe)	+	+	+ [m]	+			
tyramine	+	+				+	
tyrosine (Tyr)		–					
vitamin B1						–	–

[a] See also Fig. 2 in main text for chemical structures. [b] λ_{exc} = 400 nm, λ_{obs} = 450 nm. [c] λ_{exc} = 310 nm, λ_{obs} = 350 nm. [d] λ_{exc} = 330 nm, λ_{obs} = 370 nm. [e] λ_{exc} = 380 nm, λ_{obs} = 463 nm. [f] λ_{exc} = 310 nm, λ_{obs} = 337 nm. [g] λ_{exc} = 347, λ_{obs} = 500 nm. [h] λ_{exc} = 347, λ_{obs} = 500 nm. [i] Small changes (higher conc.). [k] Analyte was dissolved in ethanol (10 mM stock). [m] Emerging excimer band at λ_{obs} = 500 nm was monitored. [n] Control experiment in the absence of host (CB8) was also carried out. [o] λ_{obs} = 500 nm was chosen to by-pass the self-fluorescence band from the analyte. [p] Melittin is a membrane-disrupting peptide; the fluorescence response in this case is due to the release of the reporter pairs from liposomes, shifting the complexation equilibria towards the uncomplexed fluorescent dye on account of dilution.

Supplementary Methods

Calculation of the unimolecular permeation rate constants (k_p) and of the apparent permeability coefficients (P_{app}).

The passive diffusion (permeation) of small non-charged compounds follows a modified Fick's law and can be expressed by a first order kinetics.[1] Similar to first order chemical reactions, the kinetics curve can therefore be fitted monoexponentially to obtained the observed permeation rates (k_{obs}). In our assay set-up, such an exponential fit is an approximation, since it neglects that a certain fraction of transported analyte is bound to the FAR and cannot exit the liposome. Nevertheless, the fit of the kinetic curves of the phenol and aniline series (see Supplementary Table S2) with a simple monoexponential decay equation generally yielded very good fits ($R^2 > 0.95$), indicating that the approximation is justified.

In our assay, we assumed a quantitative formation of the FAR receptor (experimental concentrations: 500 μM CB8 and 550 μM dye) inside the liposome and we assumed that each analyte molecule permeating into the interior of the liposome is being complexed by the artificial receptor (strong binding) and gives rise to a signal.

The analyte-specific unimolecular permeation rate constants (k_p) can be obtained by a linear regression of the experimentally k_{obs} with $c_{analyte}$

$$k_{obs} = (k_P/c_{FAR}) \cdot c_{analyte} \qquad \text{(S1)}$$

with c_{FAR} as the concentration of the FAR receptor that is encapsulated inside the liposome. Alternatively, the initial transport rates (v_{in}) can be obtained by a linear fit of the initial part of the concentration-normalized fluorescent signal curves and k_p can be obtained as the slope when plotting v_{in} versus $c_{analatye}$.. The initial rate method may be best choice for analytes that show a strongly non-monoexponential kinetic permeation profile and whose permeation is slow enough such that the initial part of the kinetic profile is reasonably linear given the experimental signal resolution.

For a unimolecular transport process, P_{app} and k_p are in a first approximation mathematically connected by

$$P_{app} = k_p \cdot (r/3) \qquad \text{(S2)}$$

where the volume-to-surface correction factor $r/3$ accounts for the fact that the observed rates depend on the size of the liposomal assembly (characterized by radius r), and that smaller liposomes show apparently faster permeation rates.[2]

Combing equations S1 and S2, one arrives at

$$P_{app} = k_p \cdot (r/3) = k_{obs}/c_{analyte} \cdot c_{FAR} \cdot (r/3) \qquad \text{(equation 1 in main text)}$$

Dye displacement membrane assay

Dye displacement membrane assays rely on selective encapsulation of reporter pairs inside liposomes and the reversible interaction between the receptor and the translocated analyte. To conduct the assay, liposomes containing the chemosensing ensemble composed of a macrocyclic host and a dye are prepared and purified, such that a subsequently added analyte affects the dye fluorescence only if it can enter the vesicle and displaces the dye from the macrocycle. Until now, only the dye displacement has been used as the signaling mechanism for tandem membrane assays,[3, 4] whereby the competitive displacement of the dye from such host•dye complexes upon analyte addition restores the fluorescence features of the dye in solution, Supplementary Figures 13 - 14. Specifically, this signaling mechanism has been mostly employed with macrocyclic hosts with cavity space that is capable of forming 1:1 complexes, *e.g.*, cucurbit[7]uril (CB7) and the dyes berberine (BER) or palmatine (PAL), see Supplementary Figure 13a, or with *p*-sulfonato calix[4]arene (CX4) and lucigenin.[3,4] Expanding the repertoire of chemosensors for tandem membrane assays, we exploit herein also the ability of a larger macrocyclic host such as cucurbit[8]uril (CB8) to form 1:1:1 ternary complexes,[5-7] and utilize here the quencher methyl viologen (MV) in combination with the fluorescent dyes 5-hydroxy tryptophan (5HO-Trp), 2-anilinonaphthalene-6-sulfonic acid (2,6-ANS) or dapoxyl sulfonic acid, sodium salt (DapoxS), Supplementary Figure 13b. Upon addition of an analyte, the fluorescent dye, the quencher or both the fluorescent dye and quencher are displaced from the CB8 cavity, which in each case is reflected by an increase in the emission intensity of the dye (switch-on displacement assay), Supplementary Figures 14-16. Notably, upon displacement, the emission of amphiphilic 2,6-ANS and DapoxyS is likely further enhanced by their partitioning into the phospholipid membrane.

The dye displacement signal strategy has two important disadvantages compared to the preferred fluorescent artificial receptor (FAR) assays:

(*i*) The dye displacement strategy is limited to strongly binding analytes, *i.e., K*(analyte) ≥ *K*(dye), otherwise a large excess concentration of analyte is needed to generate significant emission change. For instance, non-charged analytes at 10 µM concentration such as indole were generally not suitable to displace the dyes berberine (BER) or palmatine (PAL)

from CB7. In other words, the CB7•BER and CB7•PAL are generally only applicable to analytes with a positively charged moiety such as tryptamine, but were not suitable for most analytes shown in Fig. 2 in the main text. Similarly, the membrane-encapsulated chemosensing ensemble CB8•MV•2,6-ANS can detect the permeation of indole (conc. ≥ 1 µM) but **FAR-2** is at least 10x more sensitive, see Fig. 3b in the main text. Likewise, weakly binding analytes at 10 µM analyte concentration such as *p*-cresol were unable to displace significant amounts of dye from CB8•MV•2,6-ANS.

(*ii*) The dye displacement strategy may result in apparent kinetic rates if the dye-dissociation kinetic is slow compared to the analyte permeation through the membrane. For instance, the dissociation rate of BER from CB7 was reported to be 0.8 s^{-1} at 298 K,[8] which implies that analyte-permeation kinetics that are comparably fast or faster cannot be monitored by the dye displacement approach (for instance, $k_p = 2.7 \cdot 10^6\ s^{-1}$ for phenol, section “Time-resolved analyte translocation monitoring by FARMA” in the main text & see Table 1 in the main text). Conversely, associative binding of aromatic analytes by CB7 and ternary complex formation with CB8 show rates of $>>10^6\ M^{-1}\ s^{-1}$,[8-10] in agreement with our observations for the FARs. This very fast rate of analyte-receptor binding ensures that the FARs are capable to “immediately” respond to the concentration changes resulting from analyte diffusion through the membrane, even for the fastest membrane-permeable analytes such as toluene (Table 1 in the main text).

For purely aliphatic guests that are non-detectable with the FARs, the dye displacement method remains to date the best choice, *e.g.*, see Supplementary Figures 16 & 17.

Supplementary References

1. M. Beals LG, S. Harrell. DIFFUSION THROUGH A CELL MEMBRANE. [cited 11.08.2019] Available from: http://www.tiem.utk.edu/~gross/bioed/webmodules/diffusion.htm
2. Chakrabarti, A.C. & Deamer, D.W. Permeability of lipid bilayers to amino acids and phosphate. *Biochim. Biophys. Acta - Biomembranes* **1111**, 171-177 (1992).
3. Ghale, G. et al. Chemosensing Ensembles for Monitoring Biomembrane Transport in Real Time. *Angew. Chem. Int. Ed.* **53**, 2762-2765 (2014).
4. Norouzy, A., Azizi, Z. & Nau, W.M. Indicator Displacement Assays Inside Live Cells. *Angew. Chem. Int. Ed.* **54**, 792-795 (2015).
5. Biedermann, F. & Scherman, O.A. Cucurbit[8]uril Mediated Donor–Acceptor Ternary Complexes: A Model System for Studying Charge-Transfer Interactions. *J. Phys. Chem. B* **116**, 2842-2849 (2012).
6. Biedermann, F., Vendruscolo, M., Scherman, O.A., De Simone, A. & Nau, W.M. Cucurbit[8]uril and Blue-Box: High-Energy Water Release Overwhelms Electrostatic Interactions. *J. Am. Chem. Soc.* **135**, 14879-14888 (2013).
7. Kim, H.-J. et al. Selective Inclusion of a Hetero-Guest Pair in a Molecular Host: Formation of Stable Charge-Transfer Complexes in Cucurbit[8]uril. *Angew. Chem. Int. Ed.* **40**, 1526-1529 (2001).
8. Miskolczy, Z. & Biczók, L. Kinetics and Thermodynamics of Berberine Inclusion in Cucurbit[7]uril.
   J. Phys. Chem. B **118**, 2499-2505 (2014).
9. Miskolczy, Z. & Biczok, L. Sequential Inclusion of Two Berberine Cations in Cucurbit[8]uril Cavity: Kinetic and Thermodynamic Studies. *Phys. Chem. Chem. Phys.* **16**, 20147-20156 (2014).
10. Tang, H. et al. Guest Binding Dynamics with Cucurbit[7]uril in the Presence of Cations. *J. Am. Chem. Soc.* **133**, 20623-20633 (2011).
11. Liu, S. et al. The Cucurbit[n]uril Family: Prime Components for Self-Sorting Systems. *J. Am. Chem. Soc.* **127**, 15959-15967 (2005).
12. Cao, L. et al. Cucurbit[7]uril·Guest Pair with an Attomolar Dissociation Constant. *Angew. Chem. Int. Ed.* **53**, 988-993 (2014).
13. Biedermann, F. et al. Benzobis(imidazolium)–Cucurbit[8]uril Complexes for Binding and Sensing Aromatic Compounds in Aqueous Solution. *Chem. Eur. J.* **16**, 13716-13722 (2010).
14. Bush, M.E., Bouley, N.D. & Urbach, A.R. Charge-Mediated Recognition of N-Terminal Tryptophan in Aqueous Solution by a Synthetic Host. *J. Am. Chem. Soc.* **127**, 14511-14517 (2005).
15. Rauwald, U., Biedermann, F., Deroo, S., Robinson, C.V. & Scherman, O.A. Correlating Solution Binding and ESI-MS Stabilities by Incorporating Solvation Effects in a Confined Cucurbit[8]uril System. *J. Phys. Chem. B* **114**, 8606-8615 (2010).
16. Bailey, D.M., Hennig, A., Uzunova, V.D. & Nau, W.M. Supramolecular Tandem Enzyme Assays for Multiparameter Sensor Arrays and Enantiomeric Excess Determination of Amino Acids. *Chem. Eur. J.* **14**, 6069-6077 (2008).
17. Nau, W.M., Ghale, G., Hennig, A., Bakirci, H.s. & Bailey, D.M. Substrate-Selective Supramolecular Tandem Assays: Monitoring Enzyme Inhibition of Arginase and Diamine Oxidase by Fluorescent Dye Displacement from Calixarene and Cucurbituril Macrocycles. *J. Am. Chem. Soc.* **131**, 11558-11570 (2009).
18. Ghale, G., Kuhnert, N. & Nau, W.M. Monitoring stepwise proteolytic degradation of peptides by supramolecular domino tandem assays and mass spectrometry for trypsin and leucine aminopeptidase. *Nat. Prod. Commun.* **7**, 343-348 (2012).
19. Moghaddam, S. et al. New Ultrahigh Affinity Host-Guest Complexes of Cucurbit[7]uril with Bicyclo[2.2.2]octane and Adamantane Guests: Thermodynamic Analysis and Evaluation of M2 Affinity Calculations. *J. Am. Chem. Soc.* **133**, 3570-3581 (2011).
