# Supplementary material for: Fluorescent artificial receptor-based membrane assay (FARMA) for spatiotemporally resolved monitoring of biomembrane permeability
Source: Commun Biol. 2020 Jul 15;3:383. doi: 10.1038/s42003-020-1108-9 (PMC7363885; doi:10.1038/s42003-020-1108-9)
Supplement: Supplementary file 2 — Description of Additional Supplementary Files [file 42003_2020_1108_MOESM2_ESM.pdf]

Description of additional supplementary items

Supplementary Data 1 (excel). Source data for figure 3

Supplementary Data 2 (excel). Source data for figure 4

Supplementary Data 3 (excel). Source data for figure 5
